# Embolization of high-output idiopathic renal arteriovenous fistula primarily using an atrial septal defect occluder via venous access: a case report

**DOI:** 10.1186/s12882-019-1200-x

**Published:** 2019-01-11

**Authors:** Xiaomao Chen, Qingle Zeng, Peng Ye, Hongfei Miao, Yong Chen

**Affiliations:** Department of Intervention, Nanfang Hospital, Southern Medical University, Guangzhou, 510515 China

**Keywords:** Idiopathic renal arteriovenous fistula, Atrial, Septal defect occluder, Embolization

## Abstract

**Background:**

The case report is to evaluate the efficacy and safety of embolization of a high-output idiopathic renal arteriovenous fistula (IRAVF) with an atrial septal defect occluder (ASDO) via venous access.

**Case presentation:**

A 57-year-old male diagnosed with high-output IRAVF received embolization with an ASDO via renal venous access and compact occlusion with 3 vascular plugs and a detachable elastic coil. The IRAVF was successfully occluded. After a follow-up of 2 months, renal arterial computed tomography angiography (CTA) showed the precise location of the ASDO. No complications were observed after 2 years’ follow-up.

**Conclusions:**

Based on present results, embolization of a high-output IRAVF with an ASDO via venous access might be an efficient and safe method.

## Background

Renal arteriovenous fistulas (AVFs) are uncommon malformations found in clinical practice. Idiopathic renal arteriovenous fistula (IRAVF), characterized as a single communication between the renal artery and vein, is the rarest type [1]. In recent years, interventional embolization has become the first choice for the treatment of AVFs. A prevailing embolization method is to use gelatin sponges and elastic coils to embolize fistulas through renal arterial access. However, in high-output IRAVF, these emboli have a risk of shifting. In this case, a patient diagnosed with high-output IRAVF underwent embolization with an atrial septal defect occluder (ASDO) via renal venous access, achieving a satisfactory curative effect. The detailed case report is as followed.

## Case presentation

**Fig. 1:**
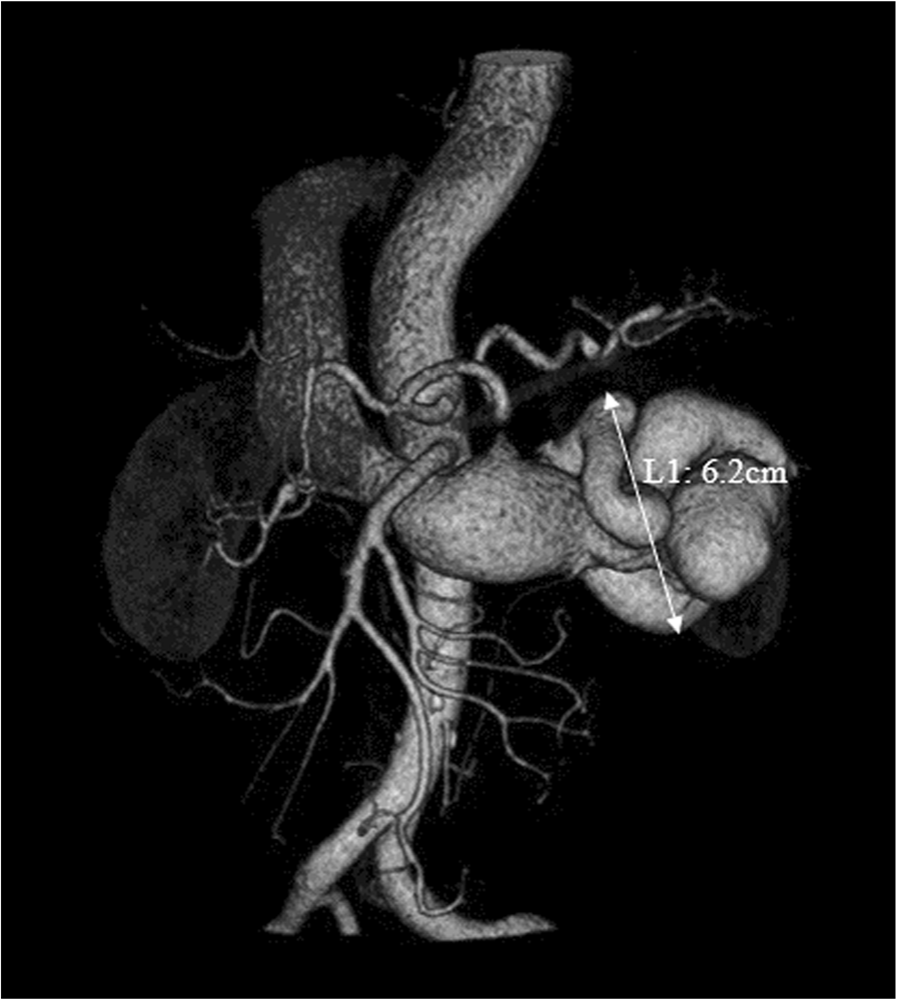
Pre-operative three-dimensional computed tomography (3D CT) reconstruction demonstrated a large left RAVF. Malformed blood vessels (L1), 6.2 cm diameter, could be clearly observed adjacent to the renal hilum

A 57-year-old Chinese man presented with repeated lumbago, chest congestion, and dyspnea on exertion for more than 6 months. The patient was found to have a left renal AVF by color Doppler ultrasonography 2 years ago. A pulsatile abdominal mass was palpable. No history of hypertension, diabetes mellitus, calculus of kidney, renal biopsy, abdominal operation or trauma. No family history. Both renal arterial computed tomography angiogram (CTA) and color Doppler ultrasonography showed a high-output IRAVF and dilated, tortuous renal artery and vein. Based on renal arterial CTA, the maximum diameter of an abnormal vascular mass in the left renal hilum was 6.2 cm and the left renal arterial trunk was 1.3 cm (Fig. 1). Ultrasound cardiogram revealed an enlarged left ventricle with myocardial hypertrophy, and a reduced left ventricular ejection fraction (LVEF) of 57%.

Puncture and catheterization were performed in the right femoral artery under local anesthesia. The fistula was shown by superselective arteriography. The guidewire was inserted into the draining vein directly through the fistula. Then, a catheter was successfully introduced into the left renal vein and the drainage vein of the fistula via the right femoral vein. The guidewire could also enter arterial end against the direction of blood flow through the draining vein of the fistula.

The right internal jugular vein was successfully punctured and a 14 French sheath was implanted after dilation. A Lunderquist ultrahard guidewire (COOK®, Bloomington, IN, USA) was delivered into the feeding arterial segment of the left renal AVF via the right internal jugular vein, inferior vena cava, and left renal vein, successively. Then, a 14 French conveying device of the ASDO (26 mm in diameter, Shanghai Shape Memory Alloy Material Co., Shanghai, China) was introduced into the draining vein of the AVF via the left renal vein. The transported ASDO was placed in the dilated segment of the drainage vein of the AVF at the point of its maximum diameter. A remarkably reduced blood flow velocity in the AVF was observed without any indications of escape of the ASDO.

**Fig. 2:**
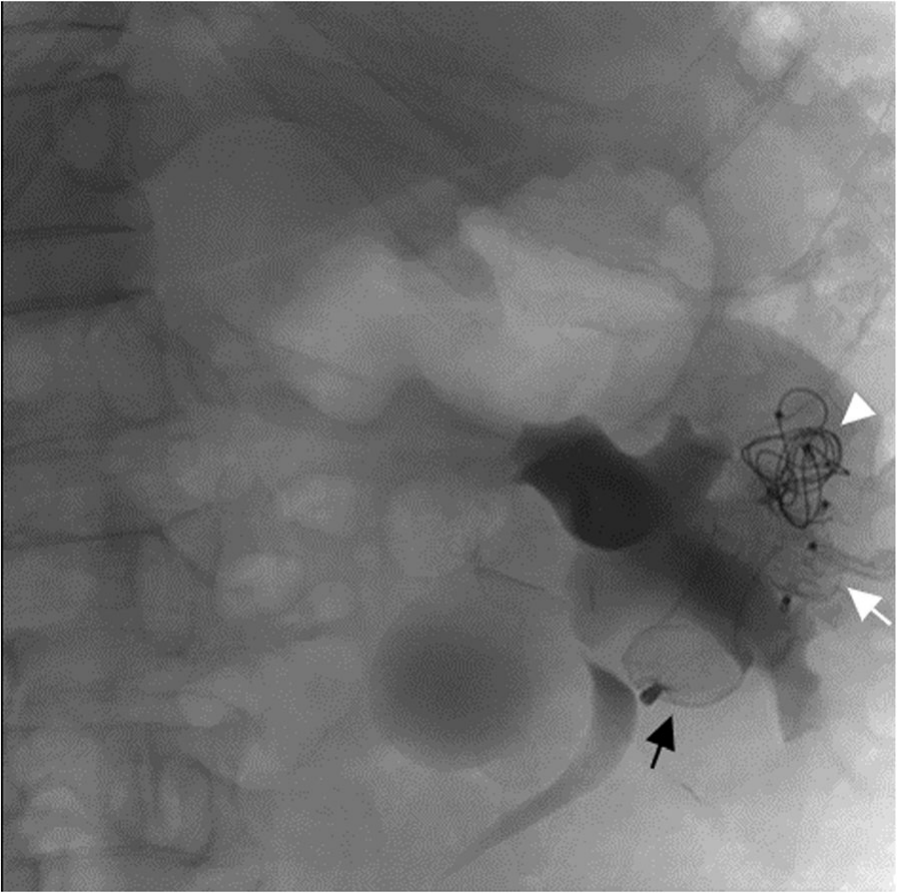
DSA showed an ASDO (black arrow), three vascular plugs (white arrow), and a detachable elastic coil (white triangle)

An 8 French guiding catheter (Medtronic, Inc., Minneapolis, MN, USA) was introduced into the drainage vein through the right femoral vein, and three vascular plugs (16 mm in diameter, AMPLATZER Vascular Plug II) were placed over the proximal end of the ASDO. Then, a 5 French Simmons catheter (Terumo LECo., Tokyo, Japan) was introduced and a detachable elastic coil (14 mm in diameter, Boston Scientific Co., Natick, MA, USA) was placed into the gap of the vascular plugs (Fig. 2). Left renal arteriography manifested that the blood flow in the AVF was stagnant, the shunt disappeared, and blood flow of the normal renal artery was unobstructed.

**Fig. 3:**
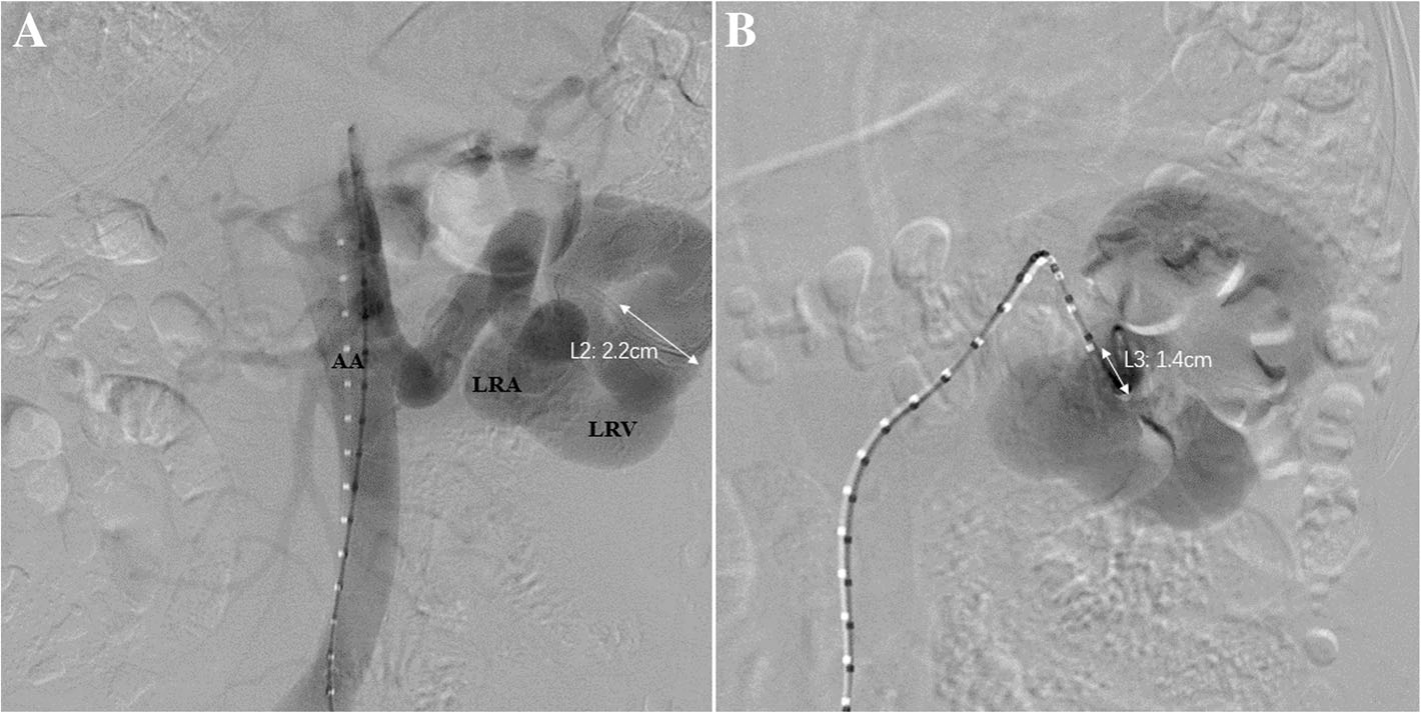
Abdominal aorta angiography demonstrated that the abdominal aorta at the upper end of the left renal artery was widened (2.2 cm in diameter). The left renal artery (LRA) was obviously thickened (1.3 cm in diameter). The left renal vein (LRV) displayed early and the maximum diameter of the dilated drainage vein was up to 2.2 cm (L2) (**a**). The guidewire was inserted into the fistula through the left renal vein and demonstrated the fistula from multiple angles. The diameter of the RAVF was measured as 1.4 cm (L3) (**b**)

Abdominal aortic angiography demonstrated an enlarged left renal arterial trunk with increased blood flow velocity and volume. A dilated branch of the renal artery was directly pouring into the drainage vein, the left renal vein, and the inferior vena cava. The diameters of the abdominal aorta and left renal artery were 2.2 cm and 1.3 cm, respectively. On left renal arteriography, the left renal artery and vein displayed almost simultaneously and the feeding artery of the fistula was uniformly dilated and tortuous. The fistula was measured as 1.4 cm in diameter (Fig. 3). The digital subtraction angiography (DSA) of the feeding artery and the drainage vein of the fistula showed that the drainage vein was tortuous and not uniformly dilated; the maximum diameter of the segment approaching the fistula was up to 2.2 cm.

The patient had no chills, fever, vomiting, or other symptoms postoperatively. No obvious abnormalities were found on urinalysis and renal function testing (creatinine, Cr: 85 μmol/L; range 53–123 μmol/L).

**Fig. 4:**
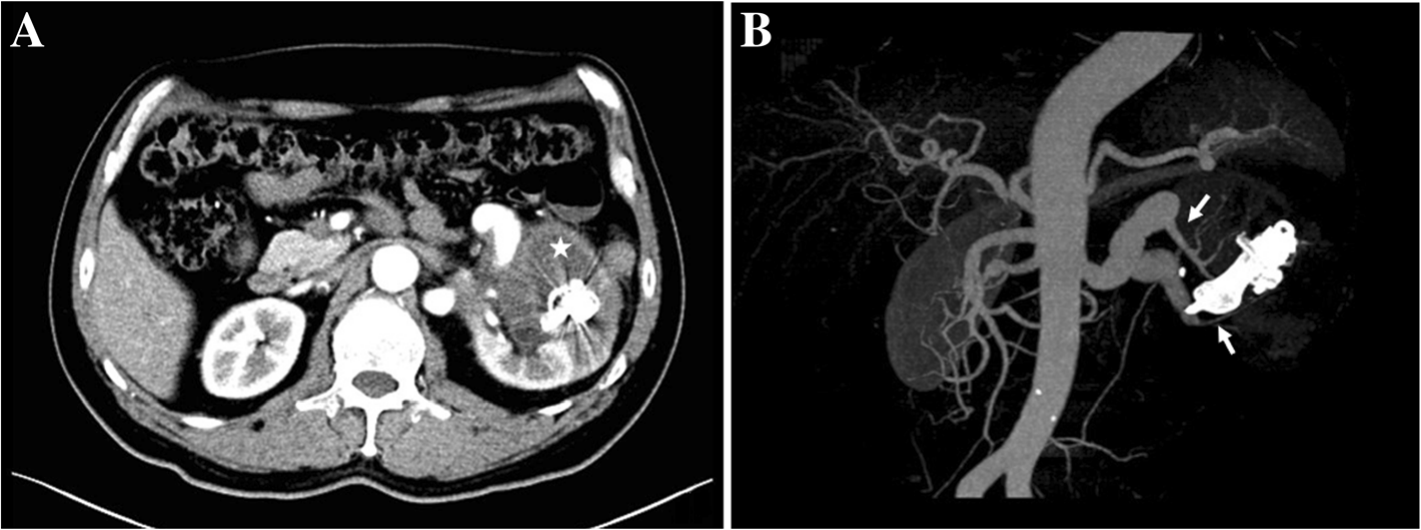
Repeat enhanced CT showed a thrombus developing in previously malformed blood vessels (white star). The residual left renal parenchyma displayed well (**a**). 3D CT reconstruction demonstrated that the normal branch of the renal artery was retained (white arrows) (**b**)

The patient’s symptoms of lumbago, chest congestion, and dyspnea on exertion completely remitted 3 months postoperatively. No obvious abnormalities were found in blood pressure and renal function (Cr: 82 μmol/L). Renal arterial CTA showed that the diameter of the renal artery reduced remarkably to 1.1 cm. Formation of intravascular thrombus within the renal AVF was observed (Fig. 4). Color Doppler ultrasonography showed that no obvious blood flow signals were found in the vasculature of the AVF. LVEF was 60% postoperatively. No postoperative complications, such as pulmonary embolism and shifting of occluders, occurred during the last 2 years of follow-up. Emission computed tomography revealed that the left renal glomerular filtration rate (GFR) was 35.3 mL/min and the right renal GFR was 23.6 mL/min. The serum creatinine was 100.3 μmol/L, the LVEF was 67% at 2 years postoperatively.

## Discussion and conclusions

Renal AVFs are uncommon malformations and typically classified as congenital (14–27%), acquired (70–80%), or idiopathic (2.8%) [2, 3]. Congenital renal AVFs usually present with multiple tortuous and dilated vessels which communicate with each other. Acquired renal AVFs are believed to be secondary to renal interventions such as surgery, trauma, biopsy, tumors, or inflammation. Whereas IRAVF is the rarest type and is hypothesized to be caused by erosion of a renal arterial branch aneurysm into an adjacent renal vein [3].

The therapeutic principle of renal AVFs is to retain functional nephrons as much as possible, while relieving symptoms and hemodynamic abnormalities. At present, the primary treatment includes surgical approaches and interventional embolization. However, surgical approaches, such as nephrectomy or ligation of the renal artery, are traumatic and cannot achieve the aim of retaining functional nephrons. In recent years, interventional embolization of AVFs with elastic coils, gelatin sponge particles, and polyvinyl alcohol particles has become a feasible, efficient, and safe method to occlude the fistula successfully while maximally retaining renal function [4]. However, for large high-output renal AVFs, there is a risk of emboli shifting to unexpected areas; these are therefore recommended for surgical treatment [5, 6]. Cao reported 28 renal AVF cases, among which three were fairly large fistulas; all of these patients preferred nephrectomy [7]. Similarly, our patient also had a high-output and extremely large fistula; the purpose of retaining sufficient blood supply of the normal renal artery could hardly be reached if the fistula was occluded via arterial access. The reasons for this are as follows. First, it is difficult to clearly manifest the position of the normal renal artery by DSA. Embolization via arterial access will increase the risk of occluding the normal renal artery. Second, on account of the risk of emboli shifting, a larger occluder was used. However, the feeding artery of the fistula was too tortuous to introduce a larger occluder to reach an ideal occlusion position. In this case, the IRAVF was successfully occluded with an ASDO via venous access. The primary advantage of this approach is that it maximally retains normal arterial blood supply and renal function. The technical key points are as follows. First, according to the results of the renal arterial CTA and angiography, we confirmed an IRAVF which had a single communication between the renal artery and vein and a swelling vein cavity (23 mm in diameter) was identified as the appropriate site to place the occluder. Second, the ASDO was chosen as the main occluder, primarily because of its larger diameter and its reliable stability of the double-disc structure. The diameter of the ASDO selected in this case was 26 mm, 30% larger than that of the target vein cavity. Third, a combination of three methods of catheterization provided a basis for satisfactory embolization.

According to previous studies, most patients suffering from high-output IRAVFs are treated by nephrectomy, especially those whose kidneys had become atrophic and in whom renal function was impaired. Nagpal et al. used vascular plugs and steel coils to embolize the renal arterial trunk [8]. The two aforementioned methods do not achieve the purpose of preserving normal nephrons. In our case, the left kidney still had normal function to some extent, therefore embolization via venous access was preferred in order to maximally retain normal nephrons. After a 2-year follow-up period, the position of occluders was appropriate, renal function was normal, and heart function improved as expected. No postoperative complications occurred. Our approach is suitable for those whose renal function has not been completely impaired and those who have appropriate positions for ASDOs.

In conclusion, embolization of high-output IRAVFs with ASDOs via venous access is a feasible, efficient, and safe method which can maximally retain normal renal function.

## Abbreviations

ASDO: Atrial septal defect occluder
AVFs: Renal arteriovenous fistulas
CTA: Computed tomography angiography
DSA: Digital subtraction angiography
GFR: Glomerular filtration rate
IRAVF: Idiopathic renal arteriovenous fistula
LVEF: Left ventricular ejection fraction
